# Probiotics and other adjuvants in allergen-specific immunotherapy for food allergy: a comprehensive review

**DOI:** 10.3389/falgy.2024.1473352

**Published:** 2024-10-10

**Authors:** Maurizio Mennini, Marisa Piccirillo, Silvia Furio, Francesco Valitutti, Alessandro Ferretti, Caterina Strisciuglio, Maria De Filippo, Pasquale Parisi, Diego Giampietro Peroni, Giovanni Di Nardo, Federica Ferrari

**Affiliations:** ^1^ Pediatric Unit, NESMOS Department, Sant’Andrea University Hospital, Sapienza University of Rome, Rome, Italy; ^2^ Pediatric Clinic, Department of Surgical and Biomedical Sciences, University of Perugia, Perugia, Italy; ^3^ Department of Woman, Child and General and Specialist Surgery, University of Campania “Luigi Vanvitelli”, Naples, Italy; ^4^ Department of Maternal Infantile and Urological Sciences, AOU Policlinico Umberto I, Rome, Italy; ^5^ Department of Clinical, Surgical, Diagnostic and Pediatric Sciences, University of Pavia, Pavia, Italy; ^6^ Section of Pediatrics, Department of Clinical and Experimental Medicine, University of Pisa, Pisa, Italy; ^7^ Pediatric Unit, Sant'Eugenio Hospital, Rome, Italy

**Keywords:** microbiome, oral tolerance, food allergy, probiotics, immunotherapy

## Abstract

This review delves into the potential of manipulating the microbiome to enhance oral tolerance in food allergy, focusing on food allergen-specific immunotherapy (FA-AIT) and the use of adjuvants, with a significant emphasis on probiotics. FA-AIT, including oral (OIT), sublingual (SLIT), and epicutaneous (EPIT) immunotherapy, has shown efficacy in desensitizing patients and achieving sustained unresponsiveness (SU). However, the long-term effectiveness and safety of FA-AIT are still under investigation. Probiotics, particularly strains of Lactobacillus, play a crucial role in enhancing immune tolerance by promoting regulatory T cells (Tregs) and modulating cytokine profiles. These probiotics can induce semi-mature dendritic cells, enhance CD40 expression, inhibit IL-4 and IL-5, and promote IL-10 and TGF-β, thus contributing to mucosal defense and immunological tolerance. Clinical trials combining probiotics with FA-AIT have demonstrated improved desensitization rates and immune tolerance in food-allergic patients. For example, the combination of Lactobacillus rhamnosus with peanut OIT resulted in a significantly higher rate of SU compared to the placebo group, along with notable immune changes such as reduced peanut-specific IgE and increased IgG4 levels. The review also explores other adjuvants in FA-AIT, such as biologic drugs, which target specific immune pathways to improve treatment outcomes. Additionally, nanoparticles and herbal therapies like food allergy herbal formula 2 (FAHF-2) are discussed for their potential to enhance allergen delivery and immunogenicity, reduce adverse events, and improve desensitization. In conclusion, integrating probiotics and other adjuvants into FA-AIT protocols could significantly enhance the safety and efficacy of FA-AIT, leading to better patient outcomes and quality of life.

## Introduction to food allergy landscape

Food allergy is a global pediatric health issue whose prevalence has risen in the past two decades. The prevalence of food allergy is currently estimated to be around 4% of children worldwide and 25% in Western countries. However, when accurately diagnosed by testing and oral food challenge, its true prevalence appears closer to 6%–8% in children, with 2.4% having multiple food allergies and 3% having experienced severe reactions (1). Standard management includes strict food avoidance, patient education, and provision of emergency medication. However, this approach is perceived as restrictive by patients, and the risk of accidental exposure still exists (1). In this scenario, allergen-specific immunotherapy (AIT) has emerged as the only treatment with disease-modifying effects (2). Allergen immunotherapy for food allergy (FA-AIT) is considered an immunomodulatory intervention for IgE-mediated food allergy based on recurrent exposure to increasing doses of food at regular intervals. This desensitization process was conceived to improve the patient's threshold to elicit an allergic reaction, reducing the risks of accidental food ingestion (3). Indeed, with promising results, FA-AIT trials started focusing on developing oral immunotherapy (OIT) for peanut, cow's milk, and hen egg allergy (4). However, in real life, FA-AIT is still not widely available for all children with food allergies, and its use is limited due to the absence of formally OIT-approved protocols in most countries. OIT usually requires months or years, and some treated patients lose tolerance once they stop taking the maintenance amount of the culprit food. In addition, it remains unclear if OIT would produce long-lasting tolerance similar to the natural tolerance acquired by previously allergic children or if it would only induce transient desensitization (5). Thus, some authors consider OIT an additional risk rather than a therapy (6).

## Managing food allergies: dietary restrictions, symptom alleviation, allergen immunotherapy and drugs

Managing food allergies has traditionally relied on dietary restrictions and symptom alleviation, but recent advancements have introduced new promising approaches (7).

### Dietary restriction

The cornerstone of managing food allergies has been strict avoidance of allergenic food. This approach is straightforward but requires meticulous effort to identify and avoid all allergen sources (8). For many individuals, particularly children, this can be challenging and significantly impact their quality of life. Accidental exposure is a constant risk, leading to anxiety and necessitating the need for emergency medication such as epinephrine auto-injectors.

It was reported that the mean number of accidental reactions is 2.10 (SD 2.0) per person per year in children managing food allergies solely through dietary restrictions. Patients who reported reactions were significantly more often women, had a significantly longer duration of food allergy and had significantly more often a confirmed allergy for peanut, sesame and vegetables compared with patients who did not report reactions (9).

While effective in preventing allergic reactions, dietary restrictions do not address the underlying immune response.

### Symptom alleviation

Symptom alleviation in food allergy management primarily involves using antihistamines and corticosteroids to manage mild to moderate allergic reactions. For severe reactions, epinephrine is the treatment of choice. These medications do not prevent allergic reactions but help manage symptoms.

While symptom alleviation is critical in acute management, it does not provide a long-term solution, and patients remain at risk of accidental exposure and subsequent reactions.

### Allergen immunotherapy

FA-AIT has emerged as a promising method for inducing tolerance to food allergens. It involves regularly administering gradually increasing amounts of the allergen to modify the immune response. There are three primary forms of FA-AIT: oral immunotherapy (OIT), sublingual immunotherapy (SLIT), and epicutaneous immunotherapy (EPIT) (10).

#### Oral immunotherapy (OIT)

OIT involves ingesting small, gradually increasing amounts of the allergen, typically in food or capsules. The goal is to desensitize the immune system to the allergen, allowing the patient to tolerate more significant amounts without experiencing severe reactions.

OIT had a significant positive effect in achieving tolerance, with a relative risk (RR) of 11.94 for peanut allergy compared to avoidance or placebo (7).

While OIT has shown promise in inducing desensitization and, in some cases, sustained unresponsiveness (SU), it is associated with a high incidence of adverse reactions, particularly gastrointestinal symptoms. These adverse events are usually mild to moderate but can impact the patient's quality of life and adherence to the therapy (11).

#### Sublingual immunotherapy (SLIT)

SLIT involves placing small doses of the allergen under the tongue, where it is absorbed into the bloodstream. This method is less invasive than OIT and generally has a better safety profile, with fewer and milder adverse reactions (12).

Despite its safer profile, SLIT may be less effective than OIT in inducing desensitization and SU.

#### Epicutaneous immunotherapy (EPIT)

EPIT involves the application of a patch containing the allergen to the skin. The allergen is absorbed through the skin, which has a high density of immune cells that can promote tolerance. EPIT is associated with mild local skin reactions, including itching and redness (13).

EPIT's non-invasive nature and favorable safety profile make it an attractive option, particularly for young children who may not tolerate OIT or SLIT well. However, its efficacy in inducing long-term tolerance is still under investigation.

### Biologics

Biologics, such as monoclonal antibodies targeting IgE, offer another avenue for managing food allergies. Omalizumab, an anti-IgE antibody, has shown promise in increasing the threshold of allergens required to trigger a reaction (14).

Biologics can be adjuncts to FA-AIT to enhance its efficacy and reduce adverse reactions. They work by lowering the levels of free IgE, thereby decreasing the immune system's sensitivity to the allergen. This approach can be particularly beneficial for patients who experience severe reactions during FA-AIT.

The efficacy of FA-AIT and biologics in achieving desensitization and SU is well-supported by meta-analysis, which shows significant positive effects compared to avoidance or placebo (7). However, the long-term efficacy and safety profiles remain areas of concern. Most adverse reactions reported during FA-AIT are mild to moderate, but there is limited data on severe or life-threatening adverse reactions.

Studies have reported significant improvements in quality of life (QoL) measures for patients undergoing FA-AIT, reflecting reduced fear of accidental exposure and, more importantly, social participation.

However, the burden of frequent clinic visits, the potential for adverse reactions, and the requirement for long-term adherence to treatment protocols can negatively impact QoL. Balancing the benefits and challenges of FA-AIT is essential to ensure that patients derive maximum benefit from these therapies (15).

Future research should focus on long-term follow-up studies to assess the durability of tolerance induced by FA-AIT and biologics.

#### Potential role of adjuvants to Fa-AIT

Adjuvants, derived from the Latin word adjuvant—meaning “to help” -are usually added to vaccines to enhance their antigen-specific immune response and reduce some undesirable reactions (16). In allergy, an adjuvant is a substance or compound co-administered with the allergen extract and can increase allergen immunogenicity and modulate the immune response (17). Their role has, therefore, been investigated in OIT to improve the duration of tolerance, allow the administration of lower doses, and reduce treatment duration and side effects (18, 19). The ideal adjuvant should be biodegradable, stable, sustainable, non-toxic, and cost-effective, able to promote an appropriate immune response and combine optimal physiochemical properties with biological activity properties (17).

Several types of adjuvants have been evaluated for FA-AIT. However, in allergy, the preexisting T_H_2 immune response is very robust, and establishing a protective immune reaction requires application schemes over 3–5 years to induce an immunomodulatory process. Although rates of successful desensitization are generally much high for SLIT and OIT, the effects are commonly lost after treatment cessation, and patients need to continue immunotherapy indefinitely to ensure ongoing tolerance (20).

Furthermore, safety concerns exist for food immunotherapy related to adverse allergic events (AEs). For EPIT and SLIT, AEs are generally limited, typically involving the treatment site, whereas OIT may provoke systemic reactions, including anaphylaxis requiring epinephrine use.

The optimal adjuvant should improve the safety and efficacy of FA-AIT by leading to faster relief of clinical symptoms and resulting in better patient adherence (21).

#### Rational of bacterial therapies

Studies have investigated bacterial therapies, such as probiotics, in treating allergic disorders, although challenges still need to be addressed due to study variability and inconsistent outcome measures. Probiotics significantly contribute to the production of Th1 cytokines, induce regulatory T cells (Tregs), and suppress Th2 pathways. Specifically, Lactobacillus strains facilitate the generation of semi-mature dendritic cells (DCs), enhance CD40 expression, inhibit IL-4 and IL-5, and promote IL-10 and TGF-β. They also boost local IgA production, which is essential for mucosal defenses (22, 23).

Research has explored the immunomodulatory effects of probiotics in promoting tolerance, showcasing their anti-inflammatory properties and ability to reduce reactive oxygen species (ROS) (24). Probiotics help balance Th1 and Th2 responses by regulating pro-inflammatory and anti-inflammatory cytokines and influencing gene expression during inflammation, which affects cell morphology and targeting. DCs, as antigen-presenting cells (APCs), are crucial for distinguishing between commensal bacteria and probiotics, with toll-like receptors (TLRs) playing a vital role in this process.

In food allergy treatment, the induction of Tregs has garnered significant interest. B regulatory cells (Bregs) exhibit immunosuppressive functions in various inflammatory diseases, including food allergies. Bregs produce cytokines such as IL-10, TGF-β, and TSP1, which repress T-cell mediated inflammation, enhance Treg function, induce tolerogenic DCs, and alter the phenotype of other local B cells. Bacterial signals can influence Breg phenotypes through interactions with short-chain fatty acids (SCFAs) like butyrate. Butyrate increases the production of the serotonin-derived metabolite 5-hydroxyindole-3-acetic acid (5-HIAA), which binds to the Aryl Hydrocarbon receptor (AhR). AhR acts as a transcriptional regulator for Bregs, boosting IL-10 production and suppressing pro-inflammatory cytokines such as TNFα and IL-6 (25).

## Key findings from clinical trials exploring probiotic supplementation alongside Fa-AIT for managing food allergies

Recent studies have explored the efficacy of combining probiotics with OIT to treat food allergies. One significant study investigated the effects of a probiotic, *Lactobacillus rhamnosus* CGMCC 1.3724, combined with peanut OIT (PPOIT) in peanut allergic children. This double-masked, placebo-controlled, randomized trial aimed to induce SU and found that 82.1% of participants receiving PPOIT achieved SU compared to just 3.6% in the placebo group. Furthermore, 89.7% of the PPOIT group were desensitized to peanuts, demonstrating significant immune changes, including reduced peanut-specific IgE levels and increased peanut-specific IgG4 levels. However, PPOIT-treated participants reported more adverse events, mainly during home dosing.

It is necessary to underline that the mentioned study compares only the PPOIT group with the placebo, without including a comparison arm for those who underwent peanut OIT alone. It is important to highlight that this represents a significant limitation of the study, as it does not allow for an assessment of whether the benefits of PPOIT are solely due to the presence of probiotics or also to the effects of OIT itself (26).

Further research assessed the impact of PPOIT on health-related quality of life (HRQoL). In a study, those who received PPOIT showed significant improvements in HRQoL measures, such as the Food Allergy Quality of Life Questionnaire (FAQLQ-PF) and Food Allergy Independent Measure (FAIM). Improvements were noted at three months and sustained up to 12 months post-treatment, indicating that PPOIT helped achieve SU and enhanced participants’ psychosocial well-being (27).

A long-term follow-up study, conducted four years after the cessation of treatment, evaluated the persistence of PPOIT's benefits. The study found that participants receiving PPOIT were significantly more likely to continue consuming peanuts and had fewer allergic reactions than the placebo group. These findings suggest that PPOIT provides long-lasting clinical benefits and persistent suppression of the allergic immune response (28).

An economic analysis of PPOIT demonstrated its cost-effectiveness, estimating the cost per quality-adjusted life year (QALY) gained to be approximately $A20,000, below the conventional value judgment threshold, suggesting it is a good value for money (29).

Another study explored the combined effects of *Lactobacillus casei variety rhamnosus* (Lcr35) and egg OIT in a mouse model of egg allergy. The results indicated that co-administration of Lcr35 and OIT significantly reduced the severity of anaphylaxis and decreased ovomucoid-specific IgE levels, effects that were sustained even after ceasing treatment. This combination also reduced mucin production in the small intestine, highlighting a synergistic effect in enhancing protection against anaphylaxis (30).

A phase IIb multicentre, double-blind, randomized, controlled trial (PPOIT-003) randomly assigned to receive probiotic (*Lactobacillus rhamnosus,* ATCC 53103) and peanut oral immunotherapy, placebo probiotic and peanut OIT, or placebo probiotic and placebo OIT for 18 months, and were followed up until 12 months after completion of treatment. During the 12-month post-treatment period, 60 (85%) of 71 participants in the PPOIT group, 60 (86%) of 70 participants in the OIT group, and six (18%) of 34 participants in the placebo group were eating peanut; rescue epinephrine use was infrequent (two [3%] of 71 in the PPOIT group, four [6%] of 70 in the OIT group, and none in the placebo group). The authors concluded that adding a probiotic did not improve the efficacy of OIT but might offer a safety benefit compared with OIT alone, particularly in preschool children (31).

In another study, heat-killed *Lactiplantibacillus plantarum YIT 0132* (LP0132) combined with low-dose cow milk (CM) OIT was tested in children with cow milk allergy. This randomized, double-masked, placebo-controlled trial found that LP0132 with OIT improved tolerance to CM and was associated with favorable immunological changes, such as increased specific IgG4 levels and decreased IL-5 and IL-9 levels (32).

These studies collectively demonstrate the potential of combining probiotics with OIT in treating food allergies, highlighting various strains such as Lactobacillus rhamnosus CGMCC 1.3724, Lactobacillus rhamnosus ATCC 53103, Lactobacillus casei variety rhamnosus (Lcr35), and Lactiplantibacillus plantarum YIT 0132. This combination approach improves immunological outcomes, induces sustained unresponsiveness, and enhances the quality of life for individuals with food allergies.

As noted, FA-AIT represents the primary therapeutic strategy. However, it appears to be insufficient and requires some adjuvants. Probiotics, particularly strains of Lactobacillus, could play a crucial role in this context (Table 1).

**Table 1:** Evidence of using probiotics in combination with allergen-specific immunotherapy for food allergy.

Study	Probiotic and OIT combination	Participants/model	Key findings	Adverse Events
Tang et al., (26)	*Lactobacillus rhamnosus CGMCC 1.3724* with Peanut OIT (PPOIT)	Children aged 1-10 years with peanut allergy	82.1% achieved sustained unresponsiveness (SU), 89.7% desensitized to peanuts, reduced peanut-specific IgE, increased peanut-specific IgG4	More adverse events, mostly during home dosing
Dunn Galvin et al., (27)	*Lactobacillus rhamnosus CGMCC 1.3724* with Peanut OIT (PPOIT)	51 participants	Significant improvements in health-related quality of life (HRQL) measured by FAQLQ-PF and FAIM, sustained up to 12 months post-treatment	Not specified
Hsiao et al., (28)	Lactobacillus rhamnosus CGMCC 1.3724 with Peanut OIT (PPOIT)	Long-term follow-up, 4 years post-treatment	Continued peanut consumption, fewer allergic reactions, smaller wheal sizes in skin prick tests, higher peanut-specific IgG4 to IgE ratios	Not specified
Kim et al., (30)	*Lactobacillus casei variety rhamnosus (Lcr35)* with Egg OIT	Mouse model of egg allergy	Reduced severity of anaphylaxis, decreased ovomucoid-specific IgE levels, sustained effects post-treatment, reduced mucin production in the small intestine	Not specified
Huang et al., (29)	*Lactobacillus rhamnosus* CGMCC 1.3724 with Peanut OIT (PPOIT)	Economic analysis	Cost-effective, estimated cost per QALY gained approximately $A20,000	Not applicable
Loke et al., (31)	*Lactobacillus rhamnosus ATCC 53103* with Peanut OIT	Phase IIb multicentre, double-blind, randomized, controlled trial with 200 children	Both PPOIT and OIT effective in inducing SU, PPOIT associated with fewer adverse events, particularly in younger children	Fewer adverse events in PPOIT compared to OIT alone
Yamamoto-Hanada et al., (32)	Heat-killed *Lactiplantibacillus plantarum YIT 0132* with low-dose Cow Milk (CM) OIT	Children with cow milk allergy (CMA)	Improved tolerance to CM, increased specific IgG4 levels, decreased IL-5 and IL-9 levels, increased gut microbiota diversity	Not specified

### Mechanisms, strategies, and future research directions for adjuvants in Fa-AIT

Food allergen immunotherapeutic research tried to focus on other adjuvants, including biologics, novel delivery vehicles, adjuvants designed to target toll-like receptor pathways, and other innovative alternatives. Furthermore, different routes of administration exist for adjuvants in FA-AIT: oral, sublingual, and subcutaneous (33).

### Oral immunotherapy adjuvants

#### Omalizumab

Anti-immunoglobulin E (IgE) monoclonal antibody (mAb) was introduced as an adjunctive therapy to reduce OIT-related allergic reactions. Omalizumab, a humanized monoclonal antibody targeting the fragment crystallizable (Fc) portion of IgE antibodies, thereby preventing mast cell and basophil activation, has shown promising results in the treatment of food allergy, both as monotherapy and as an adjuvant to OIT. Numerous studies showed an increase in threshold dose for various food allergens after omalizumab treatment, limiting AEs such as urticaria and anaphylaxis (34, 35), laying the foundations for more rapid escalation of OIT dosing. Therefore, studies about omalizumab facilitating OIT confirm that omalizumab allows safer, more rapid desensitization from milk, although without sustained unresponsiveness (SU) (33, 35–38). Similarly, studies about omalizumab-facilitated peanut OIT demonstrated a more rapid desensitization with low rates of adverse reactions, providing sustained unresponsiveness after discontinuation (39–41). In the United States, 30% of children with food allergies react to multiple foods, and the worldwide prevalence of numerous food allergies reaches 2.4% (42). In this scenario, the ability to simultaneously treat multiple food allergies makes omalizumab attractive as an adjuvant for FA-AIT. Three studies demonstrated the efficacy of omalizumab in achieving multifood desensitization, which persisted even after discontinuation, although the durability of the effect varied depending on the food. Furthermore, biologics may enable some patients to undergo OIT by allowing better management of relative contraindications, such as uncontrolled asthma, eczema, urticaria, or eosinophilic esophagitis (43–45).

#### Dupilumab

Dupilumab is a monoclonal antibody targeting the IL-4 receptor-α, thus inhibiting both IL-4 and IL-13 production. As omalizumab, dupilumab can succeed as an emerging adjuvant for OIT, and it is currently being studied for peanut allergy OIT (46).

#### Herbal therapy

Another novel approach to food allergy treatment is herbal therapy based on traditional Chinese medicine, which is thought to be effective in treating many diseases, including food allergies. Studies on peanut-allergic mice demonstrated that treatment with food allergy herbal formula 2 (FAHF-2) can reduce the frequency of adverse events, including anaphylaxis, down-regulating T_H_2 responses, thus enhancing OIT desensitization (47, 48). However, the only study on humans revealed no clinical benefit, probably due to poor adherence (49).

#### Interferon-γ

Interferon-γ (IFN-γ) is a pro-inflammatory cytokine that usually protects cells from viral infections, that can counterbalance TH2 responses while promoting T_H_1 reactions, reducing the production of IL-4 and IgE and inhibiting allergic sensitization (50, 51). Two studies evidenced successful sustained unresponsiveness or desensitization in food-allergic children treated with IFN-γ as an adjuvant to OIT (52, 53). Although the addition of IFN-γ seems to improve tolerability, studies are limited, and several side effects have been described related to IFN-γ administration to allergic patients.

#### Nanoparticles

Particle delivery systems are adjuvants that aim to facilitate the work of antigen-presenting cells by increasing the length of contact between the allergen and the patient's mucosa. Nanoparticles are delivery systems currently under research as new adjuvants in FA-AIT. They can provide an extra layer of protection for the allergen from degradation, therefore achieving high concentration at the site of action and increasing immunogenic properties. They can also prevent allergen recognition by IgE from basophils or mast cells, reducing allergenicity and, thus, the risk of adverse events (54). Given the promising immunomodulatory effects of CpG, two more recent studies evaluated the use of CpG-coated nanoparticles loaded with peanuts. CpG/peanut nanoparticles prevented anaphylaxis to oral peanut challenge, reduced Th2 cytokines, and increased IFN-γ levels in peanut-allergic mice (55). Recent studies have also investigated nanoparticles containing rapamycin, also known as sirolimus, an inhibitor of the mammalian target of the rapamycin (mTOR) pathway, known to induce antigen-specific immune tolerance. These studies proved the efficacy of rapamycin in attenuating allergic responses to food allergy (56, 57). Despite the potential intrinsic benefits of these systems, further studies to evaluate the safety profile of these compounds are still required to warrant the use of these emerging delivery systems as potential adjuvants in FA-AIT.

### Sublingual immunotherapy adjuvants

#### Purified microbial macromolecules

Common pathogens infecting the gastrointestinal (GI) tract have developed mechanisms to evade host immunity and cause infections. Vaccine vectors generated from those pathogens have been engineered to express different antigens, including food allergens. These vectors contain pathogen-associated molecular patterns (PAMPs), such as unmethylated CpG DNA, lipoproteins, and lipopolysaccharides that can activate the host immune system (58). However, creating modified heat-killed bacteria may be time-consuming and induce adverse events (gastroenteritis, sore throat, severe abdominal pain, and even anaphylaxis) while modifying pro-allergic T_H_2 responses.

Purified microbial macromolecules, such as DNA, lipopeptides, and proteins, may induce similar beneficial immune responses as whole-cell bacteria without the associated disadvantages. Bacteria and virus express toll-like receptor ligands that are PAMPS, which activate the host immune system and may modulate pre-existing immune responses. Microbial macromolecules expressing Toll-like receptor-9 (TLR9) ligands, such as unmethylated CpG oligodeoxynucleotides (ODN), are potent inducers of T_H_1 (59) and Treg (60) immunity and may determine a sustained decrease in IgE and IgG_1_, as well as an increase in allergen-specific IgG_2a_. Although TLR ligands (TLRL) can induce similar T_H_1-associated immune responses as bacteria, such as *Escherichia coli* and *Listeria monocytogenes*, it is suggested that TLRL adjuvants only direct immune responses to the co-administered allergen, inducing persistent protection and enhancing safety. Despite these promising results, host immunity varies with age in response to TLR stimulation (61, 62), with neonates and infants being less responsive (63).

### Subcutaneous and epicutaneous immunotherapy adjuvants

Lysosomal-associated membrane proteins (LAMPs) are integral membrane proteins specific to lysosomes, thought to play an essential role in the degradation of extracellular material and phagocytosis (64). A novel approach currently under investigation involves the insertion of DNA encoding the allergen in a plasmid containing the coding sequence for LAMP, thus inducing the APCs to the synthesis of an allergen-LAMP fusion protein hypothesized to elicit T_H_1 responses (65). One study is currently evaluating the tolerability, safety, and immune responses in peanut-allergic adolescents receiving intradermal injections of ARA-LAMP-vax [NCT03755713 (66)].

Aluminum salts (alum) remain the most used form of adjuvant in FA-AIT formulations in Europe, given their ability to enhance safety through a limited rate of systemic exposure (67). Alum's precise mechanism of action has not been clarified yet. However, it is believed to adsorb proteins via electrostatic interaction with the protein's hydroxyl groups (depot effect), reducing allergen diffusion, thus lowering the chance of anaphylactic reactions and prolonging the exposure of immune cells to these antigens at the injection site (68). It is a robust inducer of a T_H_2-mediated response, promoting antigen-specific Immunoglobulin (Ig) E and IgG_1_ and Interleukin-4 (IL-4), although arguably counter-intuitive for AIT (19, 69). In food allergy, aluminum hydroxide adsorbed modified peanut extract (HAL-MPE1) administered subcutaneously reduces allergic responses while retaining peanut extract's immunogenicity (PE) (70).

Protamines are arginine-rich proteins that can spontaneously assemble into nanoparticles with CpG-ODNs, which drive the immune response toward T_H_1 responses. Findings suggest that protamine-based nanoparticles with CpG-ODN counteract the allergen-induced IgE, inducing a favorable increase in allergen-specific IgG_2a_ and may be considered a novel allergen immunotherapy delivery system (71).

### Future perspectives

Synthetic peptides representing T-cell epitope sequences of food are theorized to target allergen-specific T cells without causing IgE-mediated inflammatory cell activation (72). A novel product, PVX108, conceived for intradermal immunotherapy, demonstrated a favorable safety profile in a phase 1 trial (ACTRN12617000692336) (73).

The Bruton tyrosine kinase (BTK) is a critical component in B-cell receptor signaling and the activation of mast cells and basophils via FcεRI signaling. Ibrutinib is a BTK inhibitor currently used as an anti-neoplastic treatment and is being investigated for its potential role in food allergy immunotherapy. One study demonstrated the efficacy of Ibrutinib in decreasing skin test reactivity and IgE-mediated basophil activation test (BAT) responses to peanut and tree nuts, though without a sustained response (74). Ibrutinib was considered safe and well tolerated with no severe adverse reaction; however, only a limited sample size was involved in the study. Thus, further investigation is necessary to evaluate its potential use in preventing allergic reactions.

Finally, there is preliminary evidence for the potential utility of ketotifen, an H1 anti-histamine and mast cell stabilizer that has been used to treat a variety of allergic diseases, and leukotriene receptor antagonists (LTRAs), inhibitors of the pro-inflammatory leukotrienes’ action which have proved to be effective in asthma and in reducing side effects of OIT (75). However, more extensive randomized controlled trials, with more prolonged treatment and follow-up periods, are required to explore these compounds’ concrete effectiveness and safety in food AIT.

## Research gaps and future directions

Current research on probiotics as adjuvants to FA-AIT highlights significant gaps and suggests future research directions. While existing studies provide promising insights into the efficacy of probiotics in enhancing FA-AIT outcomes, several critical gaps remain to be addressed (16). Firstly, there is a need for large-scale randomized clinical trials to validate the efficacy and safety of probiotic supplementation in diverse patient populations. Such trials require large sample sizes and precise inclusion and exclusion criteria for this therapy, specifying the individual characteristics of patients, including age, microbiota composition, and allergy profile, which are essential to optimize the synergistic effects of probiotic-AIT combinations. Additionally, the variability in probiotic strains used across studies necessitates standardization to ensure consistent results and facilitate meaningful comparisons, which needs improvement. Furthermore, determining the optimal probiotic doses for use in combination with FA-AIT is essential to maximize therapeutic benefits, minimize potential adverse effects, and achieve therapy standardization. Long-term safety evaluations are necessary to verify probiotic-AIT combinations’ durability and sustained efficacy in managing food allergies.

Future research should focus on elucidating the mechanisms by which probiotics modulate immune responses. Investigating new formulations and delivery methods to optimize clinical outcomes, considering factors such as bioavailability and stability in the gastrointestinal tract, is necessary. Furthermore, understanding the interactions between probiotics, gut microbiota composition, and host immune response dynamics is crucial, as it could provide valuable insights for personalized treatment strategies similar to those employed in other diseases.

In conclusion, probiotic supplementation offers significant promise as an adjuvant therapy in managing food allergies. Addressing current research gaps through rigorous clinical trials and mechanistic studies allows the scientific community to establish robust evidence-based guidelines for using probiotics in combination with FA-AIT. This approach enhances treatment efficacy and advances personalized medicine strategies, benefiting patients by mitigating allergic symptoms and improving long-term outcomes.
